# Bubbles in the Box: Recurrent Pneumothorax From Bronchopleural Fistula in Rheumatoid Arthritis

**DOI:** 10.1177/2324709619860555

**Published:** 2019-07-04

**Authors:** Ahmed Taha, Randa Hazam, Jim Tseng, Lusine Nahapetyan, Masoud Alzeerah, Asm Islam

**Affiliations:** 1 Texas Tech University Health Sciences Center, Amarillo, TX, USA; 2 Northwest Texas Hospital System, Amarillo, TX, USA

**Keywords:** bronchial fistula, rheumatoid arthritis, methotrexate

## Abstract

When considering rheumatoid arthritis (RA)-associated pulmonary diseases, interstitial lung disease and pleural disease are the most common RA-associated pulmonary manifestations while spontaneous pneumothorax and bronchopleural fistula (BPF) are among the extremely rare ones. To the best of our knowledge, all the previous reports of RA-associated BPFs were attributed to peripherally located pulmonary nodules that necrotized, burst into the pleural cavity, and eventually lead to the fistula formation. However, we hereby present the first case of BPF in an RA patient that formed in the absence of any underlying rheumatic pulmonary nodules. Additionally, our patient was on chronic methotrexate therapy, and there are no data in the literature that suggest methotrexate-induced parenchymal lung disease can predispose to BPF formation. Our report is the first to introduce a probe to further investigate this association.

## Introduction

Although bronchopulmonary fistulas (BPFs) are most commonly seen after lung surgeries, chronic instances are associated with infections and pleural space fibrosis.^1^ Among the chronic etiologies of BPF, rheumatoid arthritis (RA) has been associated with BPF due to its properties as a multisystem disease that can manifest as recurrent pulmonary infections, interstitial pulmonary fibrosis, and amyloidosis.^2^ Oftentimes, clinical manifestations, such as pulmonary nodules or structural defects, evolve and lead to empyema, pleural effusion, or pulmonary fibrosis before BPF appears.^3^ In this case, however, the patient had RA with no pulmonary nodules or structural defects who presented with tension pneumothorax and persistent chest tube air-leak as the primary manifestations. Due to the high morbidity and mortality of undiagnosed BPF,^4^ we are shedding light on the diagnosis of BPF in RA patients even in the absence of nodules or structural lung disease. Additionally, our patient had been taking methotrexate (MTX), hydroxychloroquine, and prednisone for decades. Hydroxychloroquine has never been reported to mediate pulmonary side effects. Although -MTX is well known for causing parenchymal lung diseases, there are no data in the literature to suggest MTX-induced parenchymal lung disease leads to BPF formation. Our report is the first in literature that introduces a probe to further investigate this association.

## Case Report

We are presenting the case of a 79-year-old Caucasian female with history of rheumatoid arthritis for 40 years and does not have prior history of established structural lung or cardiac disease. She is taking prednisolone, MTX, and hydroxychloroquine for more than 10 years. She presented to the emergency department with 2-day history of worsening dyspnea and palpitations. She was traveling across several states within the United States by a private motor vehicle; however, she did not travel to any high-altitude region. She also did not have any sick contacts. Past medical and family histories were noncontributory. In the emergency department, she was tachypneic, tachycardic, and her oxygen saturations were in the low 80s% in ambient air.

**Figure 1.**
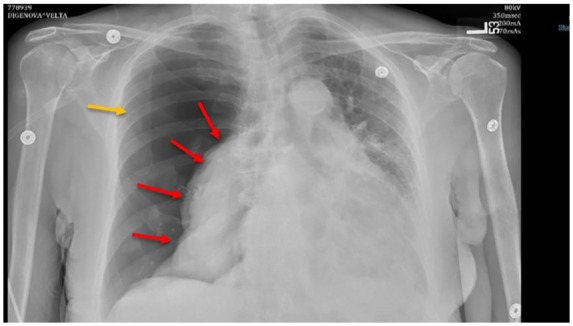
Chest X-ray showing extensive right side pneumothorax (yellow arrow) complicated by complete right lung collapse (red arrows).

**Figure 2.**
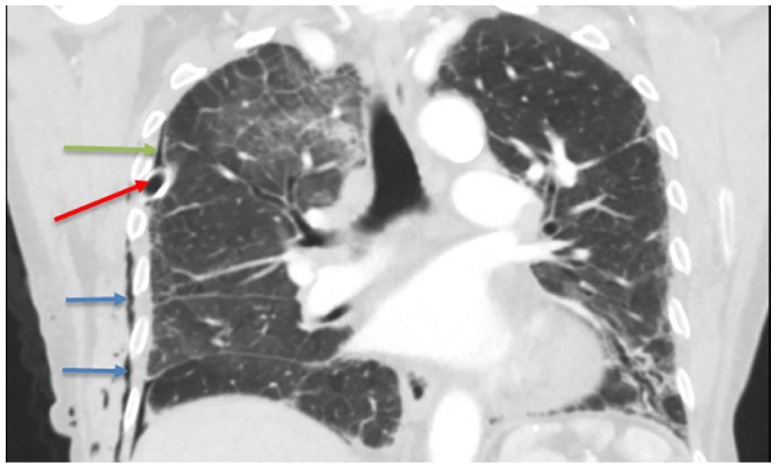
Noncontrast chest CT scan showing resolving right side pneumothorax (green arrow), mild right subcutaneous emphysema (blue arrows), right-side chest tube (placed for tension pneumothorax; Red arrow). No pulmonary nodules were seen.

Physical examination revealed bi-basilar coarse crackles, decreased breath sounds over the right hemi-thorax, and hyper-resonant percussion note. An emergent chest X-ray (CXR) showed massive right-sided tension pneumothorax with collapsed right lung (Figure 1). A right-sided 8-French surgical chest tube was inserted. A confirmatory computed tomography (CT) of the chest showed well-positioned chest tube, right pneumothorax, and no pulmonary nodules (Figure 2). Initial laboratory workup showed neutrophilic leukocytosis (28 000, 95% neutrophils), arterial blood gas: 7.52/31/58 on 2 L of oxygen. Complete metabolic panel, procalcitonin, and 3 sets of troponin I were all unremarkable. Blood, urine, sputum, and fungal cultures were negative. Transthoracic echocardiogram showed pulmonary hypertension with right ventricular pressure of 50 to 60 mm Hg.

On admission to general medical floor, the patient had good oxygen saturations on 2 L of nasal cannula, and a repeated CXR showed 90% resolution of the pneumothorax. On day 2, the chest tube was removed due to slippage into the subcutaneous layer and the formation of subcutaneous emphysema (Figure 2), and pneumothorax was confirmed to be mostly resolved. On the next day, however, a repeat CXR showed re-formation of right sided pneumothorax (occupying 40% of the right hemithorax), for which a 10-French pig tail chest tube was inserted to the right pleural space.

**Figure 3.**
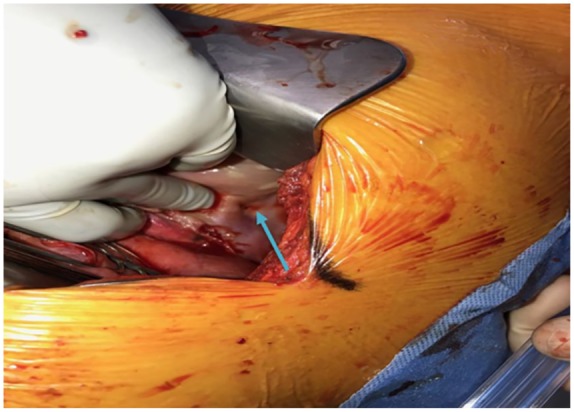
Image was taken intra-operatively showing the broncho-pleural fistula (blue arrow).

**Figure 4.**
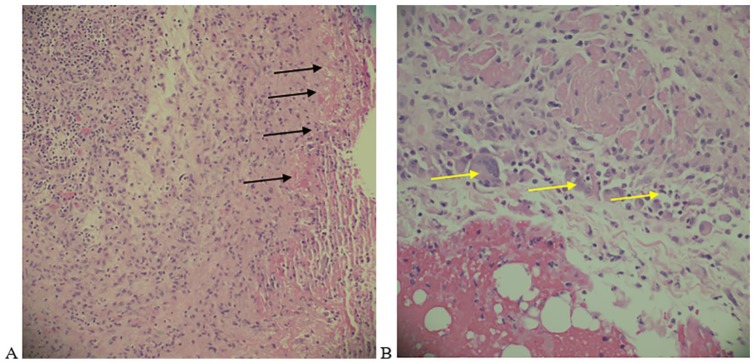
Hematoxylin and eosin section of right lower lobe wedge biopsy showing (A) fibrosing pleuritis and subpleural septal fibrosis (black arrows) with (B) acute and chronic histiocytic inflammation (yellow arrows). No pulmonary nodules were seen.

The chest tube under-water air leak persisted for 5 days following the insertion of the pig tail chest tube and the presence of BPF was suspected; therefore, an explorative thoracotomy was considered. A standard posterolateral thoracotomy and pleuritic exploration showed the presence of BPF at the apical segment of the right lower lobe (Figure 3). The BPF was sutured, covered with pleural patch, and anterior and posterior chest tubes were also inserted to the right pleural space intraoperatively. The pathological examination of right lower lobe wedge biopsy revealed fibrosing pleuritis with acute and chronic histiocytic inflammation and subpleural septal fibrosis (Figure 4). Postoperatively, the patient continued to improve, with remarkable decrease in air leak and chest tube aspirate and the chest tubes were removed in about day 10 postoperatively.

## Discussion

Approximately 1% of patients with RA develop chronic lung disease, which can lead to complications.^5^ RA-associated pulmonary manifestations include pleural effusion, pneumothorax, pulmonary infections, pneumonitis, interstitial pulmonary fibrosis, pulmonary nodules, and bronchogenic carcinoma, arteritis with pulmonary hypertension, obliterative bronchiolitis, bronchiectasis, and amyloidosis.^3,6^

On one hand, pleural involvement was described as the most common intrathoracic manifestation of rheumatoid disease, occurring in about 5% of patients.^3^ Although pleural complications in RA are related to the development of diffuse peripheral pulmonary nodules, the development of pneumothorax has only rarely been described in RA.^5,7^ Pleural effusions and pneumothorax can be caused by necrosis of a peripheral nodule leading to cavity formation that—if it bursts to the pleural space—can also lead to the formation of BPF.^8^

In our case, the pathological examination of the lung biopsy at the site of the BPF did show fibrosing pleuritis with acute and chronic histiocytic inflammation and subpleural septal fibrosis, which were correlated to long-standing RA and MTX therapy, but no pulmonary nodules were seen. This makes our case the first in the literature to report BPF formation in RA despite the absence of underlying pulmonary nodules and warrants further studies to evaluate for possible unknown pathological processes.

On the other hand, there have been a variety of drug-induced lung and pleural toxicities reported in virtually all of the disease modifying anti-rheumatic drugs (DMARDs).^9^ Although the exact pathogenesis of these toxicity is unknown, the risk and type of lung toxicity varies among the different agents used, and they include alveolar, interstitial inflammation, or fibrosis.^9,10^ In addition to direct lung toxicity, almost all the DMARDs have immunosuppressive effects that increase the risk of bacterial and opportunistic lung infections.^11-13^ Our patient was using MTX, hydroxychloroquine, and prednisone for decades.

Hydroxychloroquine has never been reported to mediate pulmonary side effects, while prednisone has been reported to rarely cause pulmonary disease through suppressing patients’ immunity and predisposing them to recurrent infections that could promote the formation of pulmonary nodules that eventually form a cavity. In 2008, a case report of a pulmonary nocardiosis was reported in a patient who was on chronic steroid therapy.^14^ However, MTX, a folic acid antagonist that inhibits cellular reproduction by causing an acute intracellular deficiency of folate coenzymes, is one of the DMARDs that are famous for causing parenchymal pulmonary diseases.^9^

Although MTX-induced pneumonitis (MIP) was described as the most common form of MTX-induced lung injury, representing 0.3% to 7.5%,^15^ other forms of parenchymal lung injury have also been associated with MTX use, such as acute interstitial pneumonia and cryptogenic organizing pneumonia.^16^ The exact pathogenic mechanism of MTX-induced pulmonary toxicity is still unclear; however, several theories for the pathogenesis of MTX-induced pulmonary toxicity have been described in the literature. MTX leads to hypersensitivity and immunosuppression, predisposing patients to repeated viral or other infections.^9,17^ It also induces injury to alveolar epithelial walls, suggesting a direct drug toxicity pathogenesis.^18^ There are no data in the literature that suggests MTX-induced parenchymal lung disease can predispose to the formation of BPF; thus, our report is the first to introduce a probe to further investigate this association.

## Conclusions

Formation of BPF is a rare RA-associated pulmonary disease and is often associated with peripheral pulmonary nodules, which open into the pleural cavity and lead to the fistula formation. However, as demonstrated in our case, BPF in RA patients can form even in the absence of underlying rheumatic pulmonary nodules. Additionally, there are no current data in the literature that suggest MTX-induced parenchymal lung disease can lead to BPF formation; our report is the first to introduce a probe to further investigate this association.
